# A novel nomogram and survival analysis for different lymph node status in breast cancer based on the SEER database

**DOI:** 10.1007/s12282-024-01591-5

**Published:** 2024-05-27

**Authors:** Lizhi Teng, Juntong Du, Shuai Yan, Peng Xu, Jiangnan Liu, Xinyang Zhao, Weiyang Tao

**Affiliations:** 1 Department of Breast Surgery, The First Affiliated Hospital of Harbin Medical University, Harbin, 150001 China; 2 Key Laboratory of Acoustic, Optical and Electromagnetic Diagnosis and Treatment of Cardiovascular Diseases, Harbin, Heilongjiang China; 3 Key Laboratory of Hepatosplenic Surgery, Ministry of Education, Harbin, Heilongjiang China; 4 NHC Key Laboratory of Cell Transplantation, Heilongjiang, China

**Keywords:** Breast cancer, Axillary lymph node metastasis, Nomogram, ITC, Micrometastases, IMLN, Survival

## Abstract

**Introduction:**

The axillary lymph node status (ALNS) and internal mammary lymph nodes (IMLN) expression associated with breast cancer are closely linked to prognosis. This study aimed to establish a nomogram to predict survival at 3, 5, and 10 years in patients with various lymph node statuses.

**Methods:**

We obtained data from patients with breast cancer between 2004 and 2015 from the Surveillance, Epidemiology, and End Results (SEER database). Chi-square analysis was performed to test for differences in the pathological characteristics of the groups, and Kaplan–Meier analysis and the log-rank test were used to plot and compare the correlation between overall survival (OS) and breast cancer specific survival (BCSS). The log-rank test was used for the univariate analysis, and statistically significant characteristics were included in the multivariate and Cox regression analyses. Finally, Independent factor identification was included in constructing the nomogram using R studio 4.2.0; area under curve (AUC) values were calculated, and receiver operating characteristic (ROC) curve, calibration, and decision curve analysis (DCA) curves were plotted for evaluation.

**Results:**

A total of 279,078 patients were enrolled and analysed, demonstrating that the isolated tumour cells (ITC) group had clinicopathological characteristics similar to those of micrometastases (Mic). Multivariate analysis was performed to identify each subgroup's independent risk factors and construct a nomogram. The AUC values were 74.7 (95% CI 73.6–75.8), 72.8 (95% CI 71.9–73.8), and 71.2 (95% CI 70.2–72.2) for 3-, 5-, and 10-year OS, respectively, and 82.2 (95% CI 80.9–83.6), 80.1 (95% CI 79.0–81.2), and 75.5 (95% CI 74.3–76.8) for BCSS in overall breast cancer cases, respectively. AUC values for 3-, 5-, and 10-year OS in the ITC group were 64.8 (95% CI 56.5–73.2), 67.7 (95% CI 62.0–73.4), and 65.4 (95% CI 60.0–70.7), respectively. For those in the Mic group, AUC values for 3-, 5-, and 10-year OS were 72.9 (95% CI 70.7–75.1), 72.4 (95% CI 70.6–74.1), and 71.3 (95% CI 69.6–73.1), respectively, and AUC values for BCSS were 77.8 (95% CI 74.9–80.7), 75.7 (95% CI 73.5–77.9), and 70.3 (95% CI 68.0–72.6), respectively. In the IMLN group, AUC values for 3-, 5-, and 10-year OS were 75.2 (95% CI 71.7–78.7), 73.4 (95% CI 70.0–76.8), and 74.0 (95% CI 69.6–78.5), respectively, and AUC values for BCSS were 76.6 (95% CI 73.0–80.3), 74.1 (95% CI 70.5–77.7), and 74.7 (95% CI 69.8–79.5), respectively. The ROC, calibration, and DCA curves verified that the nomogram had better predictability and benefits.

**Conclusion:**

This study is the first to investigate the predictive value of different axillary lymph node statuses and internal mammary lymph node metastases in breast cancer, providing clinicians with additional aid in treatment decisions.

## Introduction

Breast cancer is the most frequently diagnosed malignancy and is currently considered the second most common death-related malignancy in females [1]. Previous statistical studies have shown that 266,120 women in the United States suffer from breast cancer within one year, resulting in a 15.4% mortality rate associated with breast cancer [2].

Axillary lymph node status (ALNS) is a long-standing concern in clinical practice and research as it is one of the most important prognostic indicators of distant metastases from breast cancer [3]. Sentinel lymph node (SLN) biopsy significantly improves axillary lymph node (ALN) staging. Simultaneously, using haematoxylin and eosin (H&E) staining and immunohistochemistry (IHC), we further delineated ALNS. According to the size of the tumour lymph node metastases, we divided them into macrometastases (Nx > 2.0 mm), micrometastases (Mic > 0.2 mm; ≤ 2.0 mm) and isolated tumour cells (ITC < 0.2 mm), which were categorised by the American Joint Committee on Cancer (AJCC) Cancer Staging ManualIn. Rather than the traditional dichotomy (i.e., ALN-negative or ALN-positive), ITC and Mic introduce a semi-quantitative nodal continuum that distinguishes traditional classifications. With the incidence of ITC ranging from 7 to 19% and that of Mic ranging from 8 to 45%, each of which has a high incidence rate [4–9].

The International Breast Cancer Study Group (IBCSG) 23–01 trials suggest that axillary lymph node dissection (ALND) should be contraindicated in patients with one or more Mic (≤ 2.0 mm) in SLN  when the tumours ≤ 5 cm. Provided that the patient was treated with conventional whole-breast radiotherapy and systemic adjuvant therapy, there will be no adverse impact on survival [10]. Moreover, analogous research includes the American College of Surgery in Oncology (ACOSOG) Z0011 trial, which indicates that 1–2 SLN-positive patients can be exempted from ALND with breast-conserving surgery followed by radiotherapy and adjuvant therapy [11]. In addition, an ongoing randomised clinical trial (the SERC trial) has the potential to further compare survival disparity in non-ALNDs across ALNS; unfortunately the trial has not yet reached a conclusion [12]. Accordingly, there is a continuous controversy about the survival and treatment of patients with axillary lymph node metastases especially for ITC and Mic.

Simultaneously, internal mammary lymph nodes (IMLN), as the second most important regional lymph node in breast cancer following ALN, are well known adverse prognostic factors and are associated with a significantly lower survival rate in ILMN-positive patients [13]. Yang et al. argued that neglecting IMLN clearance and systemic radiotherapy could result in a survival benefit. Also, it has been demonstrated that systemic adjuvant therapy for patients with IMLN is still relevant for long-term survival [14, 15]. However, there are few studies on IMLN, with the associated risk factors for prognosis not yet known.

In summary, although ALN and IMLN have been investigated by numerous specialists, the strategies for surgery and adjuvant treatment remain controversial and the risk factors for survival and prognosis remain unclear. Therefore, in this study, we used the Surveillance, Epidemiology, and End Result Program (SEER) database to identify the contribution of ALNS and IMLN to breast cancer and constructed nomogram models to predict patient survival, with the final score helping clinicians decide on the appropriate treatment strategy.

## Methods

### Data source

The data source for this study was the SEER database, which is a publicly available, comprehensive database of demographic information for selected US states and counties (approximately 35% of the US population), containing millions of patients, all from 18 local cancer registries (http://seer.cancer.gov/about/overview.html). The SEER database provides details on the epidemiological characteristics of patients, stage and grade of the primary tumour, varying treatment strategies, and follow-up appointments. Following a rigorous vetting and application process, we were granted access to the SEER database data and waived the right to informed consent (reference number 14492-Nov2020) [16].

**Fig. 1:**
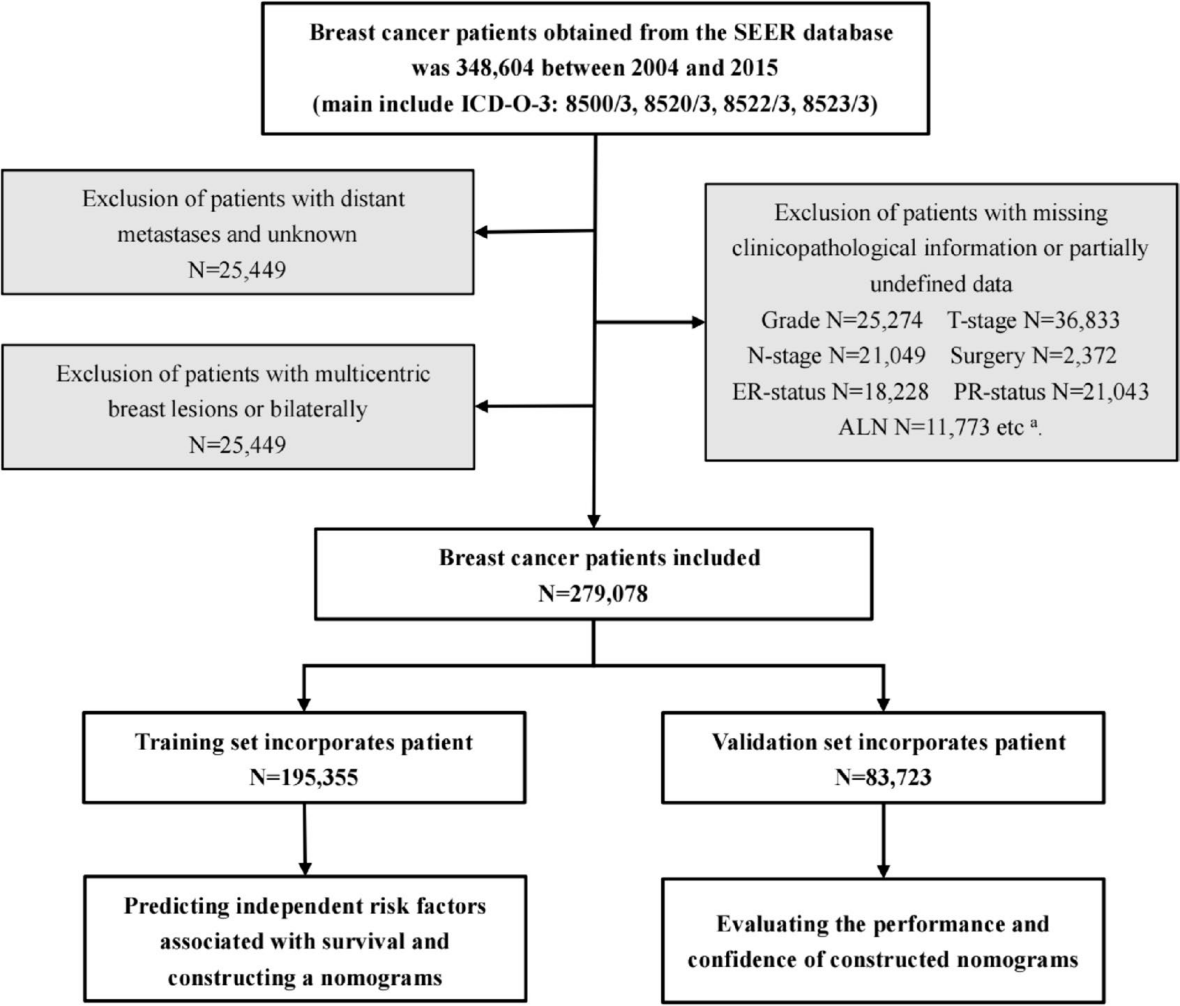
Flowchart for screening patient information derived from the SEER database. The patient details obtained contained a large number of missing data or data unsuitable for the study at the present, which also included CS size N = 21,043 and ALNS N = 15,210

We obtained demographic information from the SEER*Stat 8.4.0 software for 348,604 patients with breast cancer (the main components included ICD-O-3:8500/3, 8520/3, 8522/3, 8523/3) from 2004 to 2015. Furthermore, from the SEER, we further extracted the following: age, sex, race, grade, laterality, pathology, stage, T stage, regional nodes examined, oestrogen receptor status (ER-status), progesterone receptor status (PR-status), tumour extension, chemotherapy, radiation, surgery, ALNS, IMLN, and survival outcomes. Based on the survival data to which we had access, we further categorised patients’ survival outcomes into overall survival (OS), which is the time from the commencement of the patient's treatment strategy for any cause leading to death and breast cancer specific survival (BCSS), which is the time from the commencement of the patient's treatment until death due to breast cancer disease. The inclusion criteria were principally patients with a malignant pathological type and unilateral, primary, or single breast cancer. Moreover, the exclusion criteria were post-neoadjuvant therapy, distant metastases, recurrent tumours, multiple primary breast cancer lesions, coexistence with other tumours, and incomplete medical information. Data was collected according to the inclusion and exclusion criteria, and 279,078 people were enrolled for future evaluation and were randomly separated into a training set and a validation set (Fig. 1).

### Statistical analysis

We randomly divided the dataset into a training set, which was primarily used to construct the prediction model, and a validation set, which was primarily used for internal validation of the constructed nomogram. The applications used for the analysis and calculation of data were SPSS 22.0, with methods including the chi-square test, Kaplan–Meier method, log-rank test, univariate and multivariate analyses, and R studio 4.2.0 for the construction of nomograms (packages: rms), receiver operating characteristic (ROC) curves (packages: riskRegression and survival), calibration curves (packages: rms and survival), and decision curve analysis (DCA) curves (packages: ggDCA, rms, and survival).

The chi-square test was applied to evaluate the correlation between the training and validation sets and to verify baseline information for clinical classification. OS and BCSS curves were plotted and compared using the Kaplan–Meier method and log-rank test. The log-rank test was employed for univariate analysis, while Cox regression was utilized to construct the multivariate regression model. Hazard ratios (HRs) and 95% confidence intervals (CIs) were used for the training set. In the univariate analysis, factors with p < 0.05 were incorporated into the multivariate regression model for further analysis. Conversely, factors with p < 0.05 in the multivariate analysis were screened for nomogram construction. The predictive accuracy of the nomogram was demonstrated using a ROC curve and quantified by the area under the curve (AUC). Calibration plots were generated using the bootstrap method with 300 replications, and DCA assessed the clinical practicality and positive net benefits.

## Results

### Patients and pathological characteristics

We ultimately collected clinical characteristics from 279,078 breast cancer patients from 2004 to 2015 and randomly divided the patients into a training set (195,355) and a validation set (83,723) at a ratio of 7:3 (Supplement1). The main clinicopathological features and treatment strategies are summarised in Table 1. Among the 195,355 patients, 1295, 9236, and 1129 were in the ITC, Mic, and IMLN groups, respectively.

**Table 1:** Comparison of baseline information for breast cancer patients with different ALNS

	N0 (%)	ITC (%)	Micrometastases (%)	Nx^a^ (%)	*p*
Total	137,303 (70.2)	1295 (5.4)	9236 (24.4)	47,521	
*Age*					
< 50	25,588 (18.6)	317 (24.5)	2567 (27.8)	14,057 (29.6)	
≥ 50	111,715 (81.4)	978 (75.5)	6669 (72.2)	33,464 (70.4)	< 0.001
*Gender*					
Female	136,519 (99.4)	1286 (99.3)	9156 (99.1)	47,078 (99.1)	
Male	784 (0.6)	9 (0.7)	80 (0.9)	443 (0.9)	< 0.001
*Race*					
White	108,357 (78.9)	1002 (77.4)	7202 (78.0)	36,055 (75.9)	
Non-white^b^	28,946 (21.1)	293 (22.6)	2034 (22.0)	11,466 (24.1)	< 0.001
*Grade*					
G1	39,412 (28.7)	264 (20.4)	1903 (20.6)	5704 (12)	
G2	60,142 (43.8)	642 (49.5)	4532 (49.1)	20,665 (43.5)	
G3	37,249 (27.1)	384 (29.7)	2777 (30.1)	20,893 (44.0)	
Gx^c^	500 (0.4)	5 (0.4)	24 (0.2)	259 (0.5)	< 0.001
*Laterality*					
Left	69,632 (50.7)	647 (49.9)	4711 (51.0)	24,111 (50.7)	
Right	67,671 (49.3)	648 (50.1)	4525 (49.0)	23,410 (49.3)	0.895
*Pathology*					
Infiltrating duct carcinoma	101,978 (74.3)	850 (65.6)	7014 (75.9)	36,046 (75.9)	
Lobular carcinoma	11,343 (8.3)	207 (16.0)	782 (8.5)	4334 (9.1)	
Mixed ^d^	14,228 (10.4)	172 (13.3)	1117 (12.1)	5197 (10.9)	
Other ^e^	9475 (6.8)	65 (5.0)	304 (3.3)	1846 (3.9)	
Unknow^f^	279 (0.2)	1 (0.1)	19 (0.2)	98 (0.2)	< 0.001
*Stage*^g^					
Localized	134,037 (97.6)	1254 (96.8)	0 (0)	0 (0)	
Regional	3266 (2.4)	41 (3.2)	9236 (100.0)	47,521 (100)	< 0.001
*T-stage*					
T1	101,411 (73.9)	755 (58.3)	5274 (57.1)	16,226 (34.1)	
T2	31,447 (22.9)	447 (34.5)	3363 (36.4)	22,298 (46.9)	
T3	3401 (2.5)	87 (6.7)	483 (5.2)	6150 (12.9)	
T4	1044 (0.7)	6 (0.5)	116 (1.3)	2847 (6.0)	< 0.001
*ALN*					
< 3	80,745 (58.8)	626 (48.3)	3748 (40.6)	15,550 (32.7)	
≥ 3	56,558 (41.2)	669 (51.7)	5488 (59.4)	31,971 (67.3)	< 0.001
*ER-status*					
Positive	115,063 (83.8)	163 (12.6)	1205 (13.0)	10,031 (21.1)	
Negative	22,240 (16.2)	1132 (87.4)	8031 (87.0)	37,490 (78.9)	< 0.001
*PR-status*					
Positive	100,430 (73.1)	306 (23.6)	2107 (22.8)	15,421 (32.5)	
Negative	36,873 (26.9)	989 (76.4)	7129 (77.2)	32,100 (67.5)	< 0.001
*Tumor extension*					
Confined^h^	134,037 (97.6)	1254 (96.8)	8792 (95.2)	41,250 (86.8)	
Other^i^	3266 (2.4)	41 (3.2)	444 (4.8)	6271 (13.2)	< 0.001
*Chemotherapy*					
Yes	37,233 (27.1)	745 (57.5)	4119 (44.6)	13,076 (27.5)	
No	100,070 (72.9)	550 (42.5)	5117 (55.4)	34,445 (72.5)	< 0.001
*Radiation*					
Yes	69,874 (50.9)	657 (50.7)	4394 (47.6)	20,378 (42.9)	
No	67,429 (49.1)	638 (49.3)	4842 (52.4)	27,143 (57.1)	< 0.001
*Surgery*					
No	3475 (2.5)	4 (0.3)	26 (0.3)	1038 (2.2)	
ALND^j^	13,772 (10.0)	156 (12.0)	1992 (21.6)	25,895 (54.5)	
Non-ALND	120,056 (87.5)	1135 (87.7)	7218 (78.1)	20,588 (43.3)	< 0.001

^a^Nx mainly includes N1 patients with clinical stages of exclusion of N1mi, N2 and N3

^b^We recoded detailed race information into four major categories, the non-white population includes: Black, American Indian/Alaska Native, and Asian Pacific Islander

^c^Gx only refers to undifferentiated and anaplastic types

^d^The pathological classification is mixed, mainly including invasive ductal carcinoma and lobular carcinoma, invasive ductal carcinoma and other types of carcinoma and invasive lobular carcinoma and other types

^e^The pathological classification is other patients, its pathological types are mainly mucous adenocarcinoma, inflammatory carcinogenesis, intracystic carcinocarcinoma, tubular adenocarcinoma, adenocarcinoma, etc.

^f^Some patients have not been clinically clear or have a pathological classification missing and not undergone pathological examination

^g^Localized usually found only in the tissue or organ where it began and has not spread to nearby lymph nodes or to other parts of the body. Regional describes the body area right around a tumor

^h^In this group, the tumor is predominantly confined to breast tissue and fat, including nipples or areola

^i^In this groups, including the affected subcutaneous tissue or attached to the pectoralis major muscle or extensive skin involvement, etc.

^j^In this group, patients mainly undergo surgical procedures such as intraoperative axillary lymph node removal, including modified radical mastectomy, radical surgery for breast cancer and extended resection of breast cancer

In the training set, the median age of the patients was 60–64 years, with the majority being over 50 years (ITC: 978, 75.5%; Mic: 6669, 72.2%). Furthermore, patients were predominantly G2 in tumour grading (ITC: 642, 49.5%; Mic: 4532, 49.1%), with only a small number being G1 (ITC: 264, 20.4%; Mic: 1903, 20.6%). G4 (ITC: 5, 0.4%; Mic: 24, 0.2%) was predominantly undifferentiated, which is clinically extremely rare. In the N0 and ITC groups (N0: 134,037, 97.6%; ITC: 1254, 96.8%), most patients had localized staging; however, unlike in the Mic and Nx groups, all patients were staged regionally. There was no apparent difference in T-stage between patients in the ITC and Mic groups; however, in the N0 and Nx groups, N0 patients were mainly in the T1-stage while Nx patients were predominantly in the T2-stage. The vast majority of patients in the N0 group were positive for estrogen receptor (ER) and progesterone receptor (PR) status (ER+: 115,063, 83.8%; PR+: 110,430, 73.1%); however, a large number of patients in the ITC (ER−: 1132, 87.4%; PR−: 989, 76.4%), Mic (ER−: 8031, 87.0%; PR−: 7129, 77.2%), and Nx (ER−: 37,490, 78.9%; PR−: 32,100, 67.5%) groups tested negative. Approximately half of the patients in the ITC and Mic groups received radiotherapy and chemotherapy; however, in the N0 and Nx groups, more patients received radiotherapy, and only a small proportion opted for chemotherapy. The number of patients choosing ALND was higher in the Nx group (25,895, 54.5%) than in the N0 (13,772, 10.0%), ITC (156, 12%), and Mic (1992, 21.6%) groups. Since patients with IMLN were allocated to the Nx group, their clinicopathological features were not further described. Overall, the clinical characteristics were similar in the ITC and Mic groups, but in terms of treatment strategy, the two groups were more akin to N0 and were treated more favourably than the Nx group.

### Survival analysis and prognostic risk factors

**Fig. 2:**
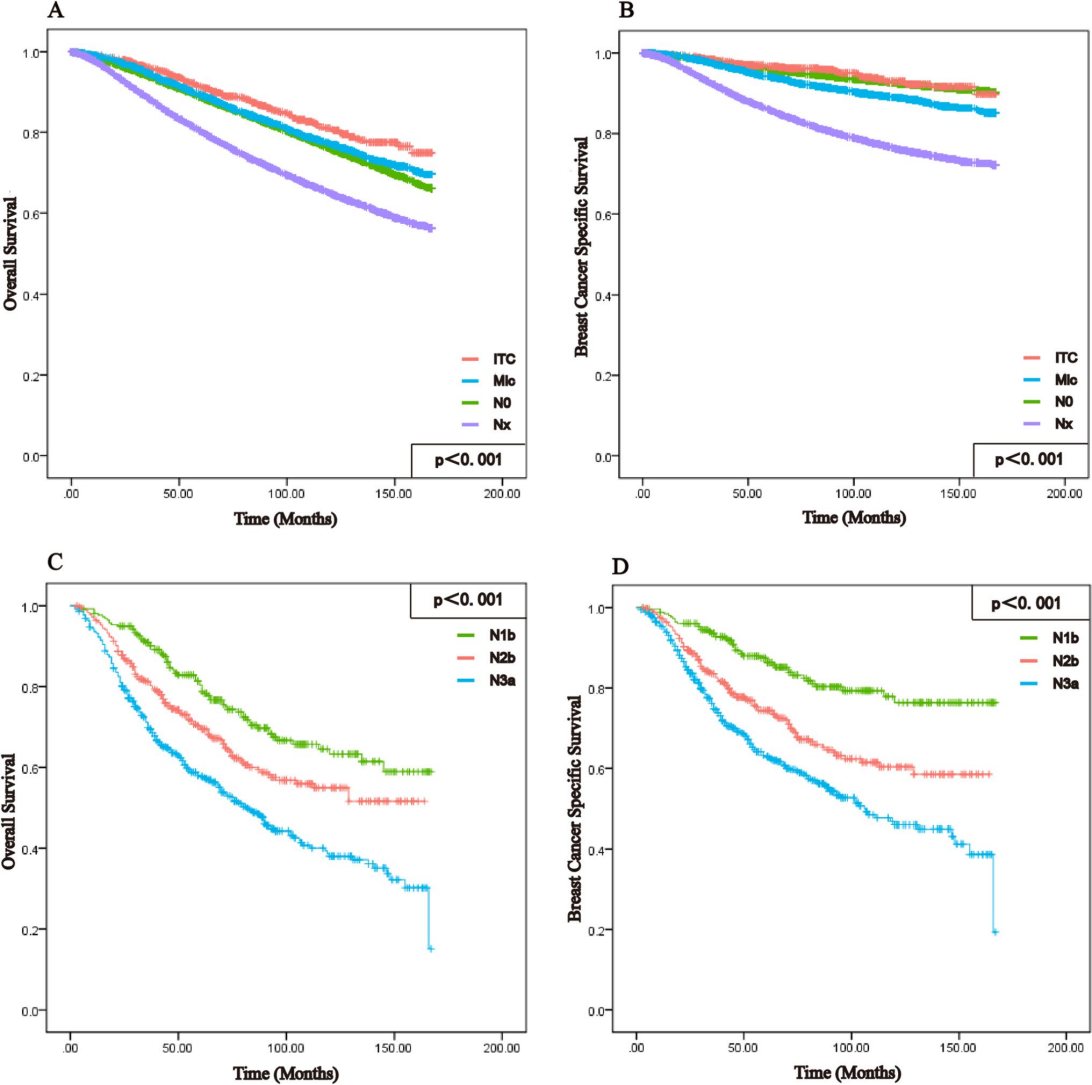
Kaplan–Meier curve were analysed for OS versus BCSS in overall patients with different ALNS and IMLN. OS **A** of total patients in the ALNS was compared with BCSS **B** for survival between groups. Comparison between OS (**C**) and BCSS (**D**) for different metastatic states in patients with IMLN

In the training set of all patients, the three-year OS was 92.8% and BCSS was 96.5%; moreover, the five-year OS was 88.3% and BCSS was 94.4%, while the ten-year OS and BCSS were 81.7% and 92.05, respectively. Furthermore, ALNS (OS: HR: 1.203, 95% CI 1.179–1.228, *p* < 0.001; BCSS: HR: 1.290, 95% CI 1.253–1.328, *p* < 0.001) was associated with OS and BCSS in both univariate analysis and multivariate Cox regression analysis (Table 2, Supplement 2), and we used Kaplan–Meier analysis to illustrate the survival differences (Fig. 2A, B, Table 3). There was no significant difference in survival curves between the N0 and ITC groups in terms of OS and BCSS, but both were higher in the OS group than in the Mic group; concurrently, patients in the ITC group had better survival than did those in the Mic group (OS: *p* < 0.001; BCSS: *p* < 0.001).

**Table 2:** Univariate and multivariate analysis conducted for all patients

	OS				BCSS			
	*p* (log rank)	HR (95% CI)	*p* (Cox)			*p* (log rank)	HR (95% CI)	*p* (Cox)
Age	< 0.001	2.271 (2.199–2.345)	< 0.001		0.001		1.284 (1.236–1.334)	< 0.001
Gender	< 0.001	0.697 (0.635–0.766)	< 0.001		< 0.001		0.851 (0.721–1.005)	0.057
Race	0.103	–	–		< 0.001		0.962 (0.928–0.997)	0.035
Grade	< 0.001	1.210 (1.191–1.230)	< 0.001		< 0.001		1.562 (1.522–1.603)	< 0.001
Laterality	0.021	0.988 (0.969–1.008)	0.253		0.081		–	–
Pathology	< 0.001	1.015 (1.004–1.026)	0.006		< 0.001		1.004 (0.987–1.022)	0.613
Stage	< 0.001	1.026 (0.965–1.090)	0.411		< 0.001		1.268 (1.160–1.385)	< 0.001
T-stage	< 0.001	1.553 (1.529–1.578)	< 0.001		< 0.001		1.748 (1.708–1.789)	< 0.001
ALN	< 0.001	0.778 (0.762–0.795)	< 0.001		< 0.001		0.834 (0.807–0.861)	< 0.001
ER-status	< 0.001	0.838 (0.810–0.867)	< 0.001		< 0.001		0.792 (0.756–0.830)	< 0.001
PR-status	< 0.001	0.783 (0.761–0.805)	< 0.001		< 0.001		0.658 (0.630–0.687)	< 0.001
Tumor extension	< 0.001	1.441 (1.379–1.506)	< 0.001		< 0.001		1.277 (1.205–1.354)	< 0.001
Chemotherapy	< 0.001	0.457 (0.463–0.487)	< 0.001		< 0.001		0.696 (0.671–0.722)	< 0.001
Radiation	< 0.001	0.612 (0.599–0.625)	< 0.001		< 0.001		0.672 (0.651–0.694)	< 0.001
Surgery	< 0.001	0.888 (0.810–0.867)	< 0.001		< 0.001		0.892 (0.864–0.922)	< 0.001
ALNS	< 0.001	1.203 (1.179–1.228)	< 0.001		< 0.001		1.290 (1.253–1.328)	< 0.001

**Table 3:** Kaplan–Meier comparing survival in individual groups

	OS				BCSS			
	Mean OS (estimated value), mo	95% CI	*p* (Log rank)			Mean BCSS (estimated value), mo	95% CI	*p* (log rank)
*ALNS*								
N0	139	139.525–140.134			158		158.142–158.534	
ITC	146	143.415–148.969			159		157.445–161.169	
Mic	141	140.435–142.658			154		153.376–155.104	
Nx	126	125.538–126.705	< 0.001		139		138.655–139.724	< 0.001

In the ITC group, the three-year OS was 96.1% and BCSS was 98.2%; furthermore, the five-year OS was 92.4% and the BCSS was 97.1%, and the ten-year OS was 87.2% and the BCSS was 95.5%, respectively. Univariate analysis was used to screen for five variables associated with OS and three variables associated with BCSS (Supplement 3), namely age (HR: 2.099, 95% CI 1.356–3.250, *p* = 0.001), chemotherapy (HR: 0.539, 95% CI 0.385–0.754, *p* = 0.001), and radiation (HR: 0.659, 95% CI 0.488–0.889, *p* = 0.006) (Table 4), while in BCSS only T-stage (HR: 1.907, 95% CI 1.351–2.692, *p* < 0.001) was determined to be associated with survival.

**Table 4:** Univariate and multivariate analysis with ITC group

	OS				BCSS			
	*p* (Log rank)	HR (95% CI)	*p* (Cox)			*p* (Log rank)	HR (95% CI)	*p* (Cox)
Age	< 0.001	2.099 (1.356–3.250)	0.001		*p* = 0.292		–	–
Grade	0.732	–	–		*p* = 0.567		1.368 (0.921–2.030)	0.120
Stage	0.276	–	–		*p* = 0.280		–	–
T-stage	< 0.001	1.229 (0.975–1.549)	0.080		< 0.001		1.907 (1.351–2.692)	< 0.001
ER-status	0.614	–	–		*p* = 0.030		0.644 (0.338–1.229)	0.182
PR-status	0.092	–	–		*p* = 0.138		–	–
ALN	0.042	0.785 (0.583–1.056)	0.110		*p* = 0.157		–	–
Chemotherapy	< 0.001	0.539 (0.385–0.754)	< 0.001		*p* = 0.535		–	–
Radiation	0.011	0.659 (0.488–0.889)	0.006		*p* = 0.646		–	–

In the Mic group, the three-year OS was 94.9% and BCSS was 97.3%; furthermore, the five-year OS was 90.6% and BCSS was 95.0%. The ten-year OS was 84.1% and 92.4%, respectively. Univariate analysis was used to ultimately screen and thirteen variables associated with OS and ten variables associated with BCSS, respectively (Supplement 4). Multivariate Cox regression analysis showed that age (HR: 2.172, 95% CI 1.881–2.510, *p* < 0.001), grade (HR: 1.292, 95% CI 1.194–1.399, *p* < 0.001), T-stage (HR: 1.426, 95% CI 1.321–1.538, *p* < 0.001), ALN (HR: 0.891, 95% CI 0.805–0.986, *p* = 0.026), ER (HR: 0.836, 95% CI 0.707–0.989, *p* = 0.037), PR (HR: 0.632, 95% CI 0.562–0.710, *p* < 0.001), tumour extension (HR: 1.568, 95% CI 1.296–1.897, *p* < 0.001), chemotherapy (HR: 0.470, 95% CI 0.422–0.524, *p* < 0.001), radiation (HR: 0.861, 95% CI 0.775–0.975, *p* = 0.006), and surgery (HR: 1.169, 95% CI 1.040–1.313, *p* = 0.009) were associated with OS and age (HR: 2.636, 95% CI 2.289–3.036, *p* < 0.001). Furthermore, grade (HR: 1.208, 95% CI 1.118–1.305, *p* < 0.001), T-stage (HR: 1.322, 95% CI 1.226–1.424, *p* < 0.001), PR (HR: 0.690, 95% CI 0.600–0.793, *p* < 0.001), tumour extension (HR: 1.649, 95% CI 1.364–1.995, *p* < 0.001), radiation (HR: 0.776, 95% CI 0.690–0.849, *p* < 0.001), and surgery (HR: 1.124, 95% CI 1.002–1.260, *p* = 0.046) was associated with BCSS (Table 5).

**Table 5:** Univariate and multivariate analysis with Mic group

	OS				BCSS			
	*p* (log rank)	HR (95% CI)	*p* (Cox)			*p* (log rank)	HR (95% CI)	*p* (Cox)
Age	< 0.001	2.172 (1.881–2.510)	< 0.001		0.013		2.636 (2.289–3.036)	< 0.001
Gender	0.019	0.757 (0.474–1.208)	0.757		0.702		–	–
Race	0.395	–	–		0.005		0.989 (0.876–1.115)	0.805
Grade	< 0.001	1.292 (1.194–1.399)	< 0.001		< 0.001		1.208 (1.118–1.305)	< 0.001
Laterality	0.014	0.911 (0.825–1.007)	0.067		0.019		0.916 (0.829–1.011)	0.082
Pathology	0.033	0.953 (0.897–1.012)	0.119		0.155		–	–
T-stage	< 0.001	1.426 (1.321–1.538)	< 0.001		< 0.001		1.322 (1.226–1.424)	< 0.001
ALN	0.027	0.891 (0.805–0.986)	0.026		0.794		–	–
ER-status	< 0.001	0.836 (0.707–0.989)	0.037		< 0.001		0.921 (0.779–1.088)	0.333
PR-status	< 0.001	0.632 (0.562–0.710)	< 0.001		< 0.001		0.690 (0.600–0.793)	< 0.001
Tumor extension	< 0.001	1.568 (1.296–1.897)	< 0.001		< 0.001		1.649 (1.364–1.995)	< 0.001
Chemotherapy	< 0.001	0.470 (0.422–0.524)	< 0.001		0.554		–	–
Radiation	< 0.001	0.861 (0.775–0.975)	0.006		< 0.001		0.776 (0.690–0.849)	< 0.001
Surgery	< 0.001	1.169 (1.040–1.313)	0.009		< 0.001		1.124 (1.002–1.260)	0.046

Across the IMLN groups, Kaplan–Meier analysis revealed a distinct difference in OS and BCSS for patients with different IMLN (Fig. 2C, D). Moreover, the three-year OS was 78.9% and BCSS was 83.0%; the five-year OS was 69.6% and BCSS was 75.6%. The ten-year OS was 60.7% and BCSS was 69.3%, respectively.

We used univariate analysis to screen for ten variables associated with OS and ten variables associated with BCSS (Supplement 5), namely age (HR: 1.268, 95% CI 1.015–1.584, *p* = 0.037), grade (HR: 1.313, 95% CI 1.118–1.543, *p* = 0.001), T-stage (HR: 1.363, 95% CI 1.195–1.555, *p* < 0.001), PR (HR: 0.689, 95% CI 0.535–0.887, *p* = 0.004), grade (HR: 0.391, 95% CI 0.310–0.494, *p* < 0.001), radiation (HR: 0.737, 95% CI 0.604–0.899, *p* = 0.003) and IMLN status (HR: 1.455, 95% CI 1.277–1.657, *p* < 0.001) were determined to be associated with OS when using multivariate Cox regression analysis. Furthermore, in BCSS grade (HR: 1.397, 95% CI 1.155–1.690, *p* = 0.001), T-stage (HR: 1.455, 95% CI 1.248–1.697, *p* < 0.001), PR (HR: 0.636, 95% CI 0.478–0.847, *p* = 0.002), chemotherapy (HR: 0.487, 95% CI 0.367–0.646, *p* < 0.001), radiation (HR: 0.778, 95% CI 0.619–0.978, *p* = 0.031), and IMLN status (HR: 1.613, 95% CI 1.380–1.885, *p* < 0.001) were determined to be associated with survival (Table 6).

**Table 6:** Univariate and multivariate analysis with IMLN metastasis group <

	OS				BCSS			
	*p* (log rank)	HR (95% CI)	*p* (Cox)			*p* (log rank)	HR (95% CI)	*p* (Cox)
Age	< 0.001	1.284 (1.028–1.603)	0.028		0.189		–	–
Gender	0.224	–	–		0.490		–	–
Race	0.071	–	–		0.026		0.852 (0.673–1.079)	0.184
Grade	< 0.001	1.343 (1.144–1.576)	< 0.001		< 0.001		1.436 (1.188–1.735)	< 0.001
Laterality	0.760	–	–		0.252		–	–
Pathology	0.772	–	–		0.904		–	–
T-stage	< 0.001	1.362 (1.194–1.554)	< 0.001		< 0.001		1.450 (1.244–1.691)	< 0.001
ALN	0.326	–	–		0.511		–	–
HR-status^a^	< 0.001	0.707 (0.576–0.868)	0.001		< 0.001		0.674 (0.537–0.846)	0.001
Tumor extension	< 0.001	1.189 (0.906–1.561)	0.211		< 0.001		1.199 (0.878–1.636)	0.254
Chemotherapy	< 0.001	0.407 (0.323–0.512)	< 0.001		< 0.001		0.507 (0.383–0.671)	< 0.001
Radiation	< 0.001	0.734 (0.602–0.895)	0.002		0.009		0.778 (0.619–0.977)	0.031
Surgery	< 0.001	1.005 (0.852–1.187)	0.951		< 0.001		1.006 (0.832–1.218)	0.948
IMLN-status^b^	< 0.001	1.460 (1.283–1.663)	< 0.001		< 0.001		1.616 (1.384–1.888)	< 0.001

^a^Hormone receptor is positive, indicating that the patient is ER-positive or PR-positive or both.

^b^Within the IMLN group there were three primary subtypes, N1b, N2b and N3a, where N1b indicates ipsilateral IMLN metastasis with ALN positivity and no obvious clinical performance; N2b indicates ipsilateral IMLN metastasis with ALN positivity and obvious clinical performance; and N3a indicates subclavian lymph node metastasis.

### Construction and validation of nomogram for predicting survival

**Fig. 3:**
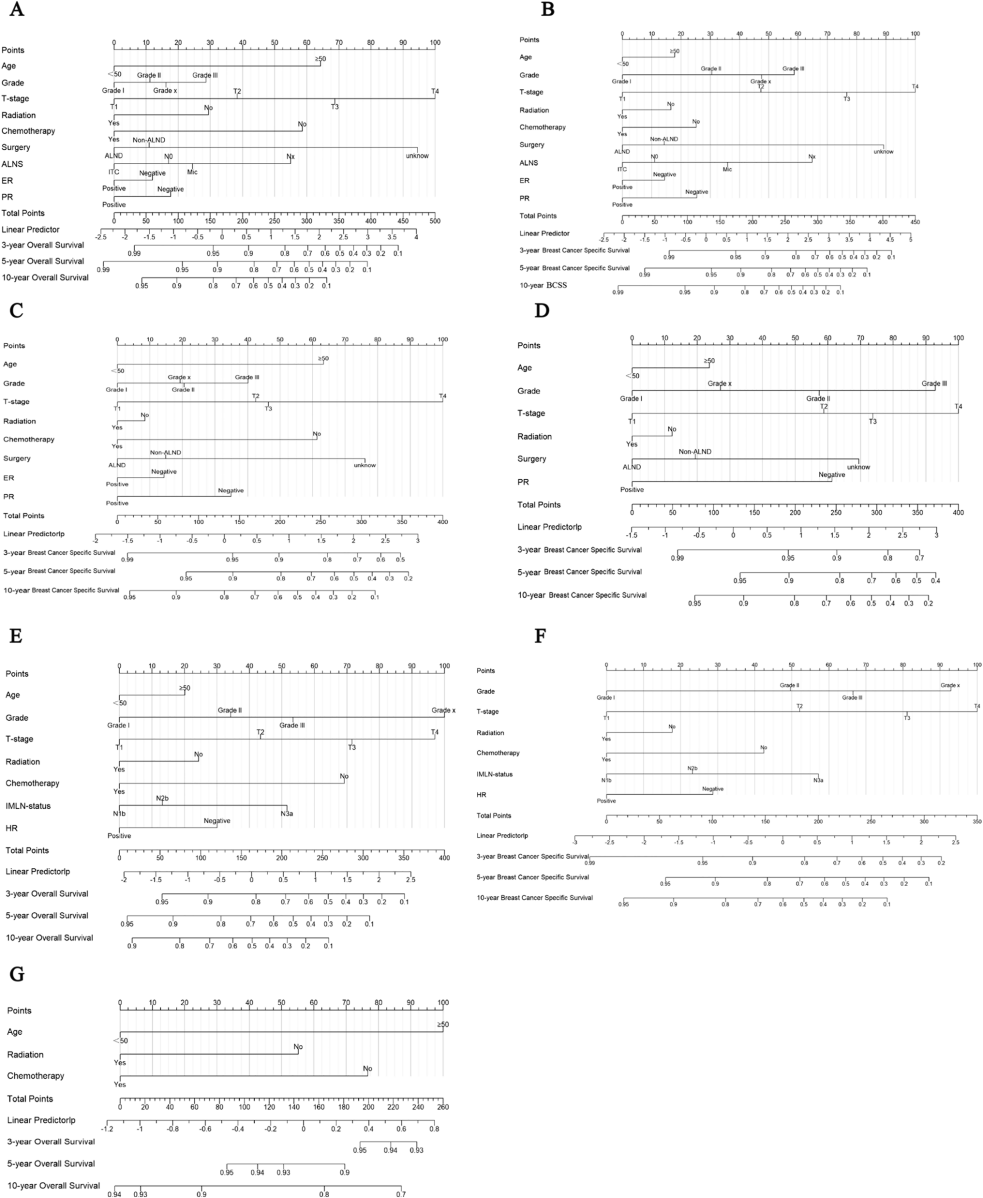
Construction of nomograms predicting 3-, 5-, and 10-years OS and BCSS in patients with ALNS versus IMLN in training set. Nomogram used to predict survival of ALNS vs IMLN. According to the patient information where each clinicopathological feature corresponds to a point at the top of the chart, the sum of all variables corresponds to a total point, and the bottom line perpendicular to the total point is the 3-, 5- and 10-years OS or BCSS. Predicting survival of OS (**A**) and BCSS (**B**) in total patients. Predicting survival of OS (**C**) and BCSS (**D**) in Mic group patients. Predicting survival of OS (**E**) and BCSS (**F**) in IMLN group patients. Predicting survival of OS (**G**) in ITC group patients

Within the training set of patients, independent prognostic risk factors associated with OS and BCSS were incorporated into the findings of clinical investigations, and multivariate Cox regression analysis was incorporated into the nomogram for 3-, 5-, and 10- year survival prediction. To better interpret the nomogram, we first drew a vertical line for each variable that corresponded to the score. Then, we added all the points and drew a vertical line over the total score to find the survival rates at 3, 5, and 10 years. The accuracy of the nomogram predictions was verified ROC, calibration, and DCA curves (Fig. 3A–G).

**Fig. 4:**
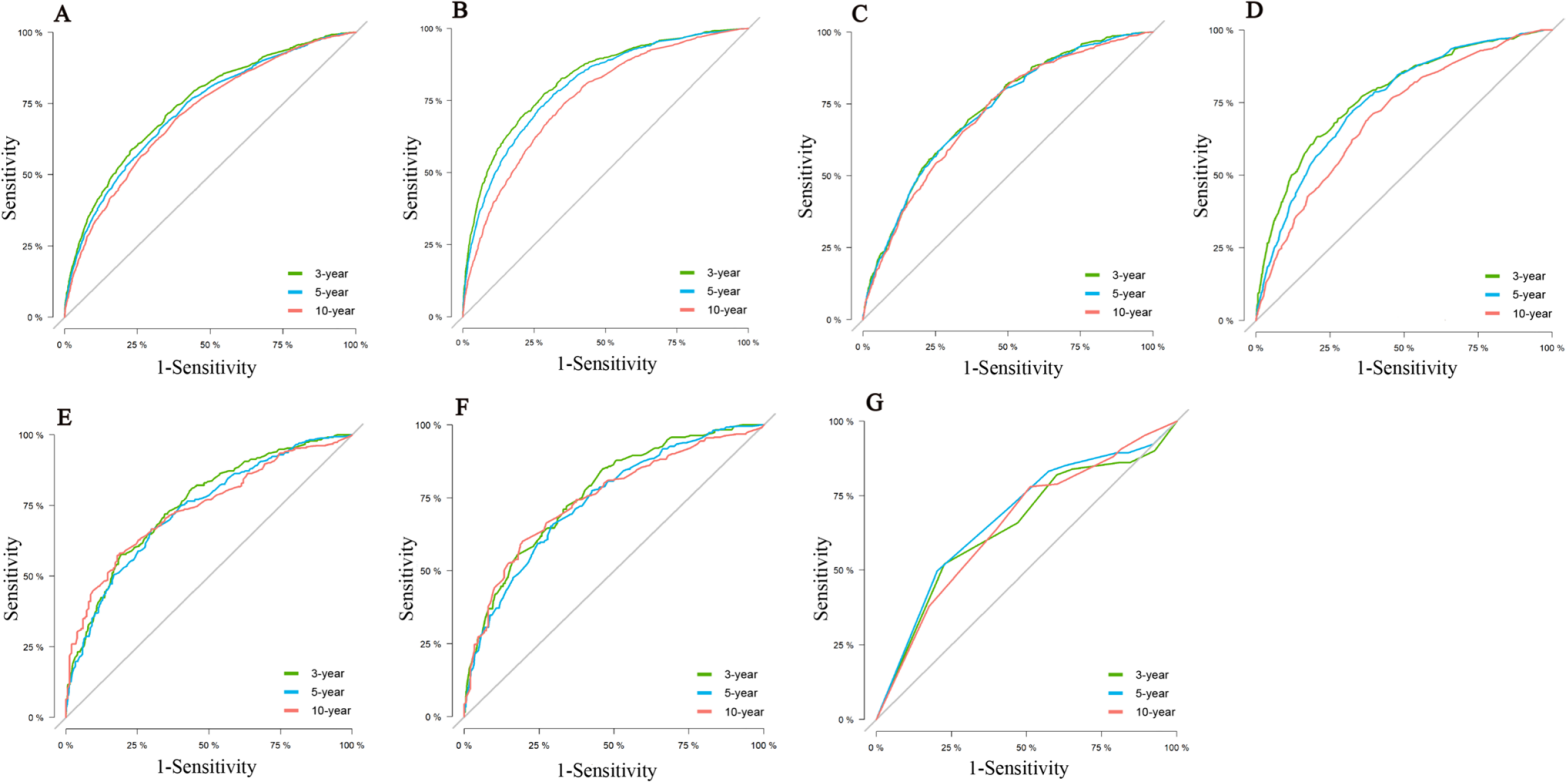
The ROC of the discriminant nomogram in training set, and the AUC values for each subset are shown in Table 7. ROC curves of OS (**A**) versus BCSS (**B**) in total patients. ROC curves of OS (**C**) versus BCSS (**D**) in Mic group patients. ROC curves of OS (**E**) versus BCSS (**F**) in IMLN group patients. ROC curve of OS (**G**) in ITC group patients

**Fig. 5:**
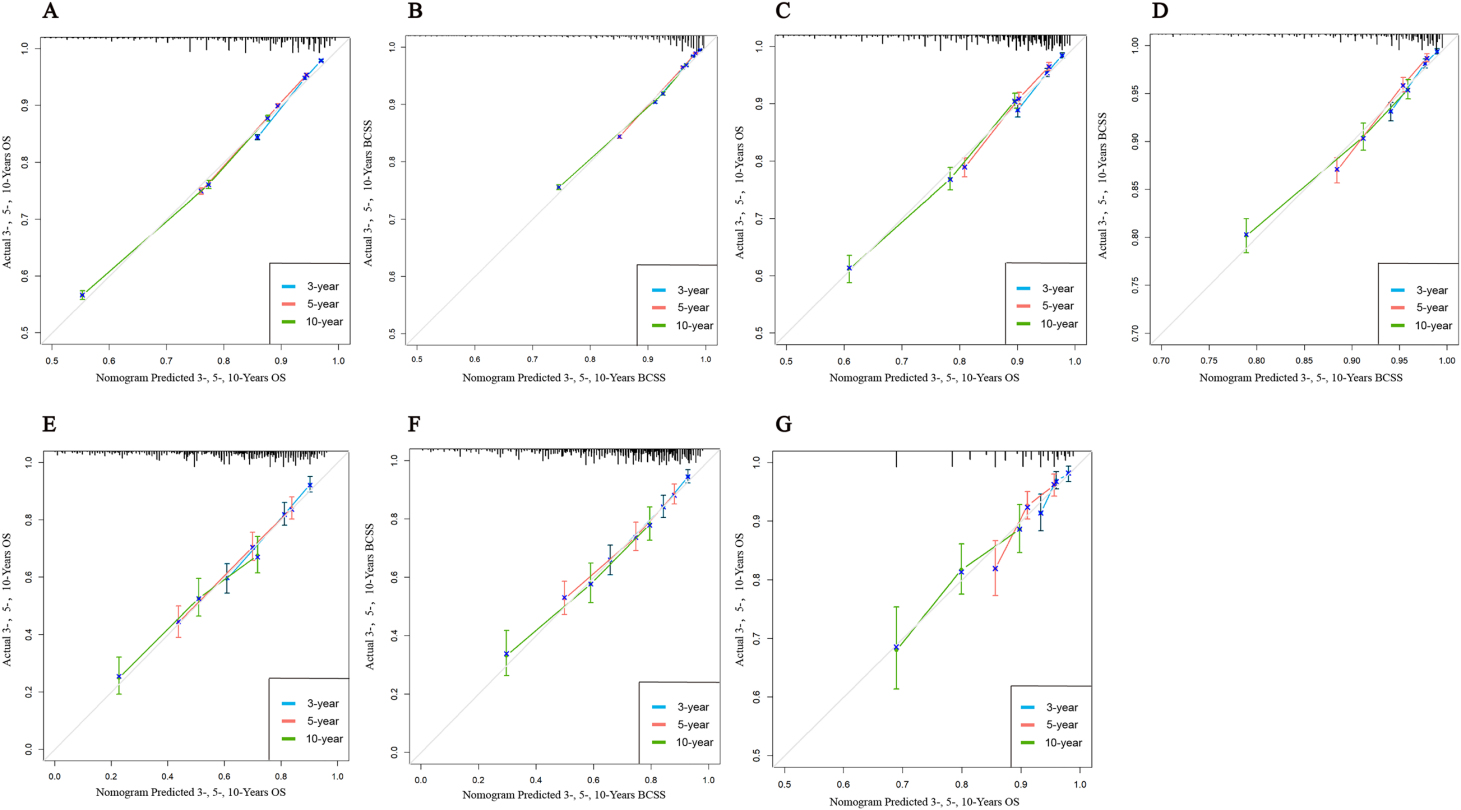
Verifying the predictive superiority of nomogram in training set, the X-axis of the calibration curve represents the predicted probability, the Y-axis represents the actual probability, and the dotted line represents the predictive ability of the calibration curve. Calibration curves of OS (**A**) versus BCSS (**B**) in total patients. Calibration curves of OS (**C**) versus BCSS (**D**) in Mic group patients. Calibration curves of OS (**E**) versus BCSS (**F**) in IMLN group patients. Calibration curve of OS (**G**) in ITC group patients

**Fig. 6:**
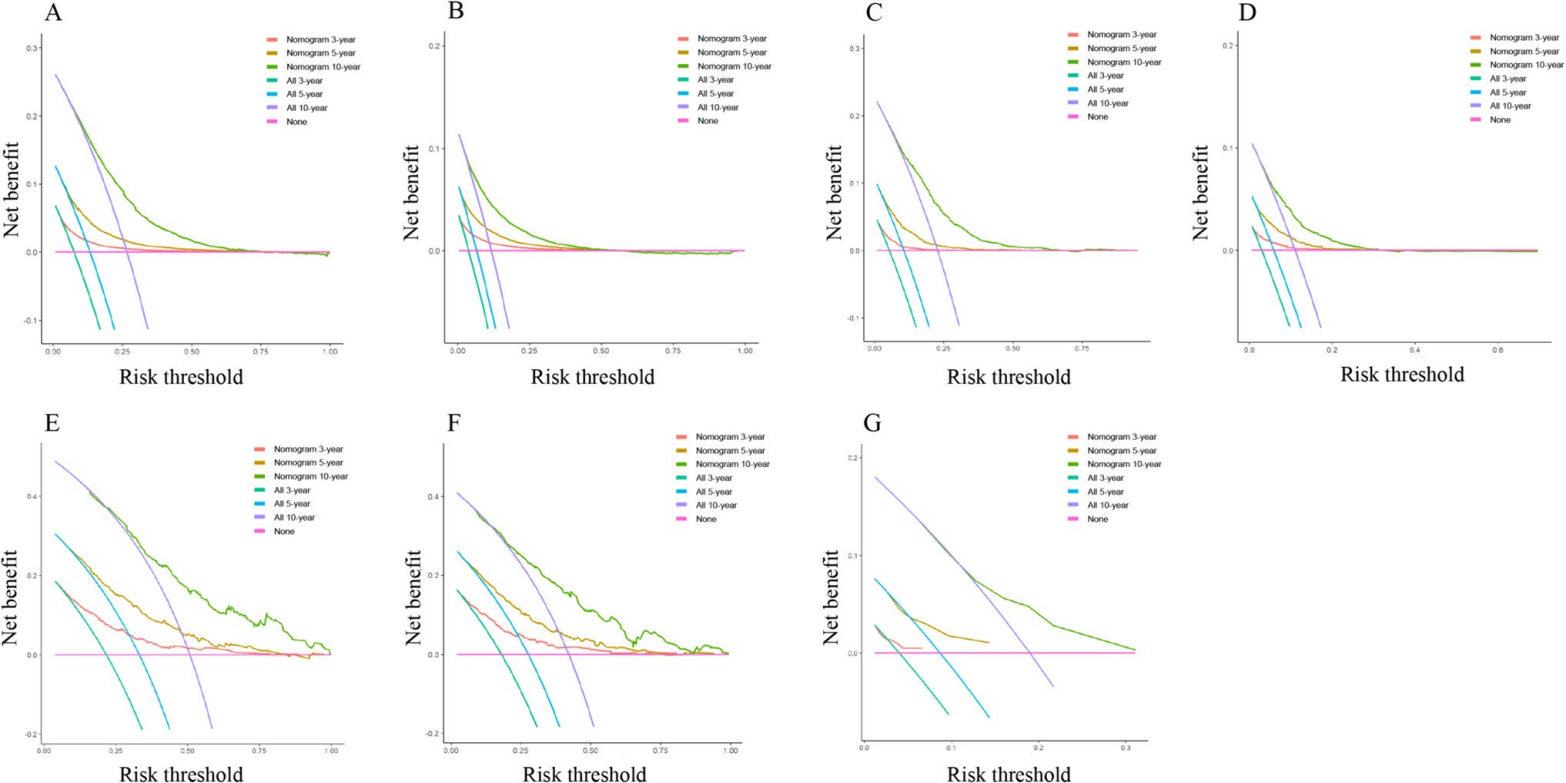
Decision curves used to predict ALNS vs. IMLN in training set, represented by the lines are shown. DCA curves of OS (**A**) versus BCSS (**B**) in total patients. DCA curves of OS (**C**) versus BCSS (**D**) in Mic group patients. DCA curves of OS (**E**) versus BCSS (**F**) in IMLN group patients. DCA curve of OS (**G**) in ITC group patients

In the training set, combining previous research and clinical practice (according to previous studies, sex, race, and side of the patient were excluded), nine variables (age, grade, T-stage, radiation, chemotherapy, surgery, ER, PR, and lymph node status) associated with OS (Fig. 3A) and BCSS (Fig. 3B) were included in the nomogram for the prediction of all patients. In the ITC group, according to clinical research and recommendations for breast cancer therapy, three variables (age, radiation, and chemotherapy) associated with OS (Fig. 3G) were included. In the Mic group, eight variables (age, grade, T stage, radiation, chemotherapy, surgery, ER, and PR) associated with OS (Fig. 3C) and six variables (age, grade, T-stage, radiation, surgery, and PR) associated with BCSS (Fig. 3D) were included. In the IMLN group, seven variables (age, grade, T stage, PR, radiation, chemotherapy, and internal mammary lymph node status) associated with OS (Fig. 3E), and six variables (grade, T stage, PR, radiation, chemotherapy, and internal mammary lymph node status) associated with BCSS (Fig. 3F) were eventually included. A Nomogram was developed to predict overall survival versus disease-free survival at 3, 5, and 10 years. Among the cohorts, the AUC values indicated a satisfactory assessment of the model, whereas the ROC curve indicated that the model had excellent clinical prediction and credibility (Table 7, Fig. 4, Supplement 7). The calibration curves showed high concordance between the predicted and actual probabilities for overall survival and disease-free survival for 3, 5, and 10 years (Fig. 5) and high concordance in the validation set (Supplement 6). The DCA curves demonstrated that the nomogram was a greater predictor of OS and BCSS for patients at 3, 5, and 10 years (Fig. 6), with high concordance in the validation set (Supplement 8).

**Table 7:** AUC from different subgroups at 3-, 5- and 10- years

	OS				BCSS			
	3-year AUC value HR(95% CI)	5-year AUC value HR(95% CI)	10-year AUC value HR(95% CI)			3-year AUC value HR(95% CI)	5-year AUC value HR(95% CI)	10-year AUC value HR(95% CI)
*Training set*								
Total patients	74.7 (73.6–75.8)	72.8 (71.9–73.8)	71.2 (70.2–72.2)		82.2 (80.9–83.6)		80.1 (79.0–81.2)	75.5 (74.3–76.8)
ITC	64.8 (56.5–73.2)	67.7 (62.0–73.4)	65.4 (60.0–70.7)		–		–	*–*
Mic	72.9 (70.7–75.1)	72.4 (70.6–74.1)	71.3 (69.6–73.1)		77.8 (74.9–80.7)		75.7 (73.5–77.9)	70.3 (68.0–72.6)
IMLN	75.2 (71.7–78.7)	73.4 (70.0–76.8)	74.0 (69.6–78.5)		76.6 (73.0–80.3)		74.1 (70.5–77.7)	74.7 (69.8–79.5)
*Validation set*								
Total patients	75.5 (74.5–76.6)	73.8 (72.9–74.7)	71.1 (70.2–72.1)		81.9 (80.6–83.2)		80.0 (78.8–81.1)	75.4 (74.1–76.6)
ITC	63.5 (52.4–74.6)	65.7 (57.3–74.2)	63.3 55.2–71.4)		–		–	*–*
Mic	77.5 (72.3–78.7)	72.5 (69.8–75.2)	70.5 (67.8–73.2)		82.6 (78.6–86.7)		76.0 (72.5–79.5)	72.7 (69.5–76.0)
IMLN	78.7 (73.7–83.8)	71.3 (65.9–76.8)	72.6 (65.7–79.5)		80.5 (74.7–86.4)		69.6 (63.2–76.0)	69.7 (61.9–77.4)

## Discussion

ALN metastasis and IMLN metastasis in breast cancer are well-known prognostic decision risk factors; moreover, ITC and Mic have a higher occurrence, but the treatment strategy and prognosis prediction for ALNS are still debated. While previous studies on ALN and IMLN have been conducted, others have had relatively limited sample volumes or lacked evidence of predictive survival [7–9, 17]. Furthermore, according to the latest available research, there are no established nomograms for predicting ALNS in breast patients. The nomogram is a simplified numerical stacking method that can assist clinicians in decision-making regarding treatment. In a retrospective review of 279,078 patients with breast cancer from the SEER database, clinicopathological characteristics, prognostic indicators, and survival were assessed, and a nomogram was constructed to predict OS and BCSS at 3, 5, and 10 years based on the results of the multivariate analysis.

Across the different nomogram prediction models, tumour stage and tumour extension were not incorporated into the construction of the nomogram because of conceptual overlap with T-staging. Similarly, in ALN, there was no clear distinction between SLN ≤ 2 or SLN ≤ 3 and the impact on clinical prognosis and treatment decisions in previous studies; thus, it was excluded in the construction of the nomogram.

Among prospective prognostic factors for patients with breast cancer, ALNS is an independent risk factor. However, Houvenaeghel et al. showed that ITC and Mic had no additional negative implications for OS and BCSS in a study of 8001 patients [18]. This contradicts our conclusion that patients with Mic had a worse prognosis than N0 patients did in terms of both OS and BCSS (Fig. 2A, B). However, no difference in survival was observed between patients in the ITC and N0 groups in this study. This is in accordance with the results of Herbert et al., who found that ITC had no adverse impact on OS or BCSS [19]. However, it is intriguing to note in Fig. 3A, B that the prediction of survival in patients with breast cancer was worse in the N0 group than in the ITC group. This may be due to several reasons: firstly, the higher number of individuals in the N0 group with more homogeneous clinicopathological features than in the ITC group; secondly, the wide time span during which lymph node biopsy techniques have improved, leading to better detection of ITC [5, 24]; finally, the fact that usually SLN ≤ 2 are performed (Table 1), which still does not exclude the possibility of misdiagnosis in the N0 group. The prognosis of ALN in breast cancer varies, particularly in mice, distinguishing between N0 and Nx. This difference necessitates the development of a more standardized treatment [9].

Prior experience with the ITC group is limited, and the therapeutic approach is usually consistent with that of the N0 group. One study demonstrated no difference in the recurrence-free survival between patients in the ITC group who underwent ALND and those in the non-ALND group [20]. Concordant with the outcomes of our study, ALND did not improve the OS and BCSS of patients, while adjuvant treatment was found to improve the OS of patients. A study by Maaskant-Braat et al., through a multivariate analysis of 9038 collected patients, demonstrated that receiving systemic therapy when ITC was present (OR 1.5, 95% CI 1.05–2.15) or Mic (OR 10.7, 95% CI 8.56–13.27) could enhance patient survival. Additionally, receiving adjuvant therapy increased the 5-year BCSS of patients, a finding that is consistent with our conclusion [21, 22]. Previous studies have suggested that age and tumour size are correlated with ITC prognosis. In contrast to the present study, in which age and tumour size (*p* = 0.08) were still considered important prognostic factors for OS and BCSS, we only found a correlation with tumour size (Table 4) [23]. Therefore, to study BCSS, we needed a broader sample to construct the nomogram.

Substantial evidence of Mic as a significant risk contributor has been demonstrated in previous research, as illustrated in the study by Yvette Andersson et al., comparing 5-year specific survival with BCSS in the N0 group (94.1% *v* 96.9% and 79.6% *v* 87.1%) [24]. Chen et al. also performed a multivariate analysis of patients with Mic using the SEER database and found that sex, ER, PR, lobular histology, grading, age, and T stage were significant prognostic risk factors [25]. Furthermore, Hetterich et al. compared adjuvant chemotherapy in 540 patients with Mic and discovered that adjuvant chemotherapy administered to the N0 group versus the Mic group did not improve OS or BCSS [5]. Compared with the present study, in addition to our evidence of the therapeutic benefit of chemotherapy in both OS and BCSS, we refined our prognostic hazard indicators and predicted OS and BCSS for patients based on clinicopathological characteristics and varying therapeutic regimens using a nomogram.

The most controversial area is whether micrometastases deserve ALND, as opposed to other ALNS. One study has shown that in cases of mice, exemption from ALND increases the 5-year recurrence rate [7]. Houvenaeghel et al. also demonstrated that waiving ALND increased recurrence and mortality following prolonged follow-up and that the procedure's conditions needed to be rigorously managed [27]. Meanwhile, studies by A. Suyoi et al., Viviana Galimberti et al., and Tsai-Wei Huang et al. have indicated that dispensing with ALN is secure for mice with a light tumour burden. Hennigs A et al. also demonstrate that the incidence of ALND is decreasing when the criteria for inclusion in ACOSOG Z0011 are met. Accordingly, we identified a preference for ALND in patients with a high axillary burden [28–32]. Furthermore, Collins et al. described how lymph node oedema from ALND far exceeds the recurrence rate [33]. In the aforementioned study, we also concluded that ALND could be eliminated in mice. First, adjuvant therapy has a higher value than ALND does, for which chemotherapy is particularly crucial. Second, in the nomogram, we discovered that ALND reduces the survival rate, which may be due to severe postoperative complications (Fig. 3C, D).

With regard to lymph node metastasis in the breast, the inevitable implication of IMLN metastasis was shown by Qi et al. to be significantly more pronounced in patients with positive ALN; however, the effect of IMLN on OS and BCSS was not found [34]. With regard to therapeutic aspects, it only proved that the prognosis of clinically diagnosed IMLN metastases was better than that of distant and IMLN metastases detected by fine-needle aspiration biopsy [35, 36]. Notably, some specialists have used mathematics to predict the prognosis of patients with IMLN metastases [37]. In contrast, our review focused more on the outcomes of patients with IMLN and quantified the importance of radiotherapy and chemotherapy in conversion to IMLN treatment. We demonstrated the significance of surgery in both OS and BCSS on univariate analysis (Table 6), but no relationship with survival was found on multivariate analysis, principally because fewer than 10 patients with IMLN metastases underwent IMLN dissection for statistical analysis. Therefore, further investigations are needed to determine the survival benefit of IMLN dissection for patients.

ALNS as a significant independent prognostic risk factor for breast cancer has been of major concern to scholars. Nevertheless, there is a paucity of available research on ITC, Mic and IMLN, as well as a lack of uniformity in treatment and long-term prognosis in the clinic. The present review constructs a nomogram about diverse ALNDs for a new therapeutic reference strategy for breast cancer patients that present with lymph node metastases. For example, for patients with Mic, which the treatment is more controversial, clinicians can apply the nomogram to predict long-term OS and BCSS in accordance with the patient's actual condition. With survival prediction, combined with clinical realities, it assists the clinician in making a decision about whether this patient should be treated with step-up or step-down therapy. An individualised therapeutic strategy for patients to maximise clinical survival benefit and achieve nomogram application.

However, there is still a limitation in the current study. First, the different molecular subtypes of breast cancer play an important role in determining the prognosis of patients; nonetheless, in the present study, due to database limitations, we were only able to retrieve data on ER and PR, while we could not further discuss HER-2 overexpression. Second, owing to the sample of patients in the ITC group itself, the ITC patients included in the SEER database all presented relatively well-characterised clinicopathology and survival outcomes, with greater survival rates, resulting in an AUC value that did not reach the best value interval in the final validation of the nomogram. Finally, it is interesting to note that we found that hormone receptor positivity in all subgroups indicated a better prognosis; however, it was not feasible to ascertain whether they benefited from endocrine therapy because of the limited information on treatment in the database. Consequently, we provide clinicians with a reference for managing different lymph node states, although there are still aspects that need to be addressed by clinical patients for further research.

## Data Availability

The datasets generated during and/or analysed during the current study are available in the SEER database repository, Surveillance, Epidemiology, and End Results Program (cancer.gov).
